# When Alterations in Social Cognition Meet Subjective Complaints in Autism Spectrum Disorder: Evaluation With the “ClaCoS” Battery

**DOI:** 10.3389/fpsyt.2021.643551

**Published:** 2021-08-26

**Authors:** Shasha Morel-Kohlmeyer, Alix Thillay, Sylvie Roux, Isabelle Amado, Lindsay Brenugat, Isabelle Carteau-Martin, Charlotte Danset-Alexandre, Baptiste Gaudelus, Jérôme Graux, Elodie Peyroux, Zelda Prost, Marie-Odile Krebs, Nicolas Franck, Frédérique Bonnet-Brilhault, Emmanuelle Houy-Durand

**Affiliations:** ^1^Centre Universitaire de Pédopsychiatrie, CHRU de Tours, Centre d'Excellence Autisme et Troubles du Neuro-développement-Tours exac.t, Tours, France; ^2^UMR 1253, iBrain, Université de Tours, Inserm, Tours, France; ^3^Institut de Psychiatrie (CNRS GDR 3557), groupe de travail ≪ Cognition ≫, Paris, France; ^4^Se Rétablir 37, CHRU de Tours, Tours, France; ^5^GHU-Site Sainte Anne, Paris, France; ^6^Centre Ressource de Remédiation Cognitive et Réhabilitation Psychosociale, île de France, Paris, France; ^7^Pôle Hospitalo-Universitaire PEPIT, GHU Psychiatrie et Neurosciences - Hôpital Sainte Anne, Paris, France; ^8^Inserm Institut Paris Neurosciences et Psychiatrie (IPNP)-UMR 1266, Paris, France; ^9^Centre Ressource de Réhabilitation Psychosociale, Centre Hospitalier Le Vinatier, Bron, France; ^10^Pôle Hospitalo-Universitaire ADIS, CRMR GénoPsy, Centre d'Excellence Autisme et Troubles du Neurodéveloppement iMind, Lyon, France; ^11^Dispositif de Soins de Réhabilitation Psycho-Sociale, Centre Psychothérapique de l'Ain, Bourg-en-Bresse, France; ^12^UMR 5229 CNRS & Université Lyon 1, Lyon, France

**Keywords:** Autism Spectrum Disorder, adults, social cognition, assessment, subjective complaints

## Abstract

**Background:** Deficit in social communication is a core feature in Autism Spectrum Disorder but remains poorly assessed in classical clinical practice, especially in adult populations. This gap between needs and practice is partly due to a lack of standardized evaluation tools. The multicentric Research group in psychiatry GDR3557 (Institut de Psychiatrie) developed a new battery for social cognitive evaluation named “ClaCoS,” which allows testing the main components of social cognition: Emotion Recognition, Theory of Mind, Attributional Style, and Social Perception and Knowledge. It further provides an assessment of subjective complaints in social cognition.

**Methods:** We compared the social cognition abilities of 45 adults with Autism Spectrum Disorder without intellectual disability and 45 neurotypically developed volunteers using the “ClaCoS” battery, in order to determine its relevance in the evaluation of social cognition impairments in autism. A correlational approach allowed us to test the links between subjective complaints and objectively measured impairments for the different components of social cognition.

**Results:** As expected, the Autism Spectrum Disorder group showed deficits in all four components of social cognition. Moreover, they reported greater subjective complaints than controls regarding their social abilities, correlated to the neuropsychological assessments.

**Conclusion:** The “ClaCoS” battery is an interesting tool allowing to assess social impairments in autism and to specify the altered components, for a better adjustment of tailored social cognition training programs. Our results further suggest that people with Autism Spectrum Disorder have a good social cognitive insight, i.e., awareness into social cognitive functioning, and may thus benefit from social cognitive training tools.

## Introduction

### Social Cognition in Autism Spectrum Disorder

Autism Spectrum Disorder (ASD) is a neurodevelopmental disorder characterized by deficits in social functioning and restricted, repetitive patterns of behavior, interests, or activities (1).

Individuals with ASD experience daily challenges with social function, which frequently impact negatively their relationships and access to education or employment. Typically, children and adults with ASD encounter difficulties to understand other's emotional states or intentions. This may lead to inappropriate behaviors in social situations. Thus, despite good intellectual abilities, high functioning adults with ASD may ask inappropriate questions, act oddly and are therefore vulnerable to bullying and isolation. These difficulties have been linked to well-documented deficits in social cognition in ASD (2). Social cognition refers to the cognitive processes (i.e., emotional processing, interpretation, mentalizing…) which allow us to adapt our responses according to different social situations, in a flexible manner (3). The heterogeneity of ASD population explains that some people on the spectrum may lack very basic social skills [emotion perception, difficulty to make eye contact…; e.g. (4, 5)] while others are mainly impaired at higher cognitive levels [mentalizing, understanding jokes, or sarcasm…; e.g. (6, 7)].

Research on social cognition in ASD has essentially focused on emotional processing (i.e., identification of emotions from different cues, including facial expression) and theory of mind (i.e., ToM; ability to mentalize other's mental states which allow us to make inferences on their intentions, beliefs, and knowledge), showing alterations in both processes (8, 9). More precisely, considerable evidence suggested deficits in the discrimination and recognition of basic emotional facial expressions [(10–13); for reviews, see (14, 15)]. However, several studies failed to show poorer recognition of emotions in ASD, especially when considering adolescents or adults without intellectual disability, although daily life difficulties were still encountered e.g. (16–21). The type of stimuli used may explain this discrepancy: deficits in the recognition of emotional expressions are more consistently observed when facial emotional processing is made more difficult by the use of complex or subtle emotions (different intensities, dynamic faces…) compared to the presentation of more basic stimuli (static faces, basic emotional expressions, only high intensity…) [(10–13); for a review, see (14)]. In particular, studies manipulating the levels of emotional intensity reported lower emotional recognition abilities in children and adults with ASD without intellectual disability. These alterations appear more strongly when using mild affective expressions (22–27).

An influential theory considering social dysfunction in ASD puts forward an alteration of ToM [Mindblindness theory, (6)]. ToM deficits in ASD have been widely supported although results in the literature remain controversial [for a meta-analysis, see (28)]. Numerous studies show a delayed or incomplete development for ToM in children with ASD e.g. (8, 29). Moreover, better performance of children with ASD in ToM tasks has been associated with better social competence (30). The results are less consistent when considering high functioning adults with ASD (31, 32). ToM deficits in this population are best revealed by tasks which mirror the demands of real life social exchanges, such as “implicit” ToM tasks, tasks which consider response times or generally more ecological tasks (33, 34). This suggests that adults with ASD succeed in classical evaluations of ToM by using compensatory strategies to infer other people's mental state (35). This is in line with functional neuroimaging studies, which show that adults with ASD activate different brain regions when solving theory-of-mind tasks (36). However, these strategies may not be efficient enough in everyday life situations, which require to instantly understand other's intentions or feelings and to respond accordingly in a timely manner (37).

Moreover, social cognitive deficits in ASD could be a consequence of an atypical sensory processing. Indeed, a great number of studies using eye-tracking have shown different visual exploration in this population, with fewer gaze directed toward social comparatively to non-social elements [(38, 39); for meta-analyses, see (40, 41)]. Moreover, children and adults with ASD benefit less than neurotypical controls from supportive contextual cues in order to recognize emotional expressions (42, 43). These observations may be explained by a lack of interest for social information (44). It could also be linked to the bias toward local vs. global information (45, 46). This results in a difficulty to perceive visual elements as a whole, even more so when considering complex visual scenes. As a consequence, ASD individuals do not sufficiently process the salient visual features which are needed in order to understand typical social situations. Altogether, these results are consistent with deficits in social perception in ASD, although this term is not commonly used in this field of research.

The alterations in the perception and understanding of social situations may lead to misinterpretations and in some adults with ASD to attribution biases. Research in this domain is scarce. Nevertheless, the few studies conducted showed no self-blaming pattern but rather healthy and normal self-serving attributions (47–49). Blackshaw et al. (47) suggest that paranoid symptoms, clinically observed in some adolescents and adults with ASD, may be a consequence of their confusion in understanding subtle social interactions and social rules. This disability could be linked to a deficit in ToM and in social perception. It may also stem from past traumatic experiences such as bullying.

### Social Cognition Evaluation in ASD

Most tools developed for neuropsychological evaluation of Emotional Processing present static photographs of highly expressive emotional expressions (e.g., Ekman Face Test) and often fail to show any impairment in ASD adults without intellectual disability (14). Indeed, it has been suggested that these adults have developed explicit cognitive, language-mediated or perceptual compensatory strategies. These strategies allow them to succeed in such classical neuropsychological evaluations of emotional perception (17, 35, 50). This further stresses the relevance of using more sensitive and ecological tools in order to assess emotional perception in this specific population.

Concerning Theory of Mind, the most classical tasks (i.e., false belief tasks; strange stories tasks…) do not sufficiently target subtle impairments encountered by adults with ASD without intellectual disability and especially in those with good verbal skills (7, 34, 51–53). These traditional tests either present static images or are based on verbal descriptions of social situations and may be solved by the use of a deductive rather than a spontaneous strategy (35, 37). Interestingly, some more ecological tasks have been developed in order to assess subtle ToM difficulties (33, 54). Among these, the MASC test has proven sensitive to reveal ToM alterations in adolescents and adults with ASD (55–60). It consists in the presentation of a short movie picturing a typical social situation (i.e., a Saturday night dinner with four young adults), thus providing a better ecological validity. Furthermore, it allows detecting hyper- or hypo-mentalizing tendencies, in addition to general ToM alterations.

To our knowledge there are very few standardized evaluation tools allowing the assessment of social perception deficits in ASD, and none of these have been standardized in French language. For instance, the Social Perception subtest from the Advanced Clinical Solution has been used to reveal such deficits in adolescents and adults with ASD (61), but is available only in English (62).

Similarly, only few standardized questionnaires have been developed in order to evaluate attribution biases (63). These questionnaires show some limitations, such as presenting few ambiguous situations with strong social cognitive biases. Moreover, they generally target paranoia and persecutory delusion rather than more general social cognitive biases. The latter might be observed in ASD but are rarely considered in clinical practice.

### The “ClaCoS” Battery: A New Tool for Social Cognition Evaluation

Deficits in social cognition are observed in several psychiatric conditions other than ASD (schizophrenia, ADHD, bipolar disorder, anorexia…), and have been linked to functional outcome [e.g., (64–67); for reviews see (68, 69)]. In the field of schizophrenia, several programs which target specific social cognition deficits have shown significant improvements in real-world outcome. This raises an important issue concerning the assessment of social cognition in order to apply the most adapted treatment for each patient. To reach that goal, a group of experts have sought to specify the definition of social cognition in order to improve its assessment in clinical practice [the SCOPE project: Social Cognition Psychometric Evaluation; (70)]. They considered social cognition as a heterogeneous construct which includes several dimensions. Pinkham (70), in accordance with Green (71, 72), proposed four main core domains: [1] Emotion processing (i.e., identification of emotions from different cues, including facial expression); [2] Social perception (i.e., decoding and interpreting social cues by taking into account the social context) and social knowledge (i.e., knowledge of social rules, roles, and goals); [3] Theory of mind (i.e., ToM; ability to mentalize other's mental state which allow us to make inferences on their intentions, beliefs and knowledge); [4] Attributional style (positive or negative inference of events). Although these different dimensions are considered and assessed separately for both theoretical and practical reasons, they may not be independent from each other but rather partially overlapping (73).

Only few validated and standardized tools are available in French language for the clinical assessment of social cognition in adults (74). In this context, the multicentric Research group in psychiatry GDR3557- Institut de Psychiatrie (www.institutdepsychiatrie.org) developed a new battery for social cognition evaluation named “ClaCoS,” which offers the advantage of exploring all four dimensions of social cognition identified by the SCOPE project. It further includes an evaluation of subjective complaints in these same four dimensions, which allows a comparison between subjective complaints in social functioning and objective evaluation. This social cognition battery has primarily a functional purpose, as it provides a singular profile of the patient's strengths and weaknesses in social cognition. This can further lead to an adjustment of tailored social cognition training programs. This battery can also be used in clinical practice, in line with a transnosographic view of social cognition deficits in different psychiatric conditions. Moreover, the “ClaCoS” battery includes ecological tools which could be of interest to assess subtle social cognition deficits encountered by adults with ASD without intellectual disability.

The aim of the present study was to examine the relevance of the “ClaCoS” battery for the assessment of social cognition in adults with ASD without intellectual disability compared to control typical developing subjects. Based on the existing literature, we expected the ASD group to be less efficient than the controls on the neuropsychological evaluation of all dimensions of social cognition, and in particular emotional perception, social perception and theory of mind. Concerning attributional style, this hypothesis could be more uncertain, as previous studies showed no difference between ASD and controls (75). Nevertheless, we predicted some differences in light of the paranoid symptoms which are clinically observed in some adults with ASD. Self-insight is typically considered to be altered in ASD (53). However, research on self-knowledge in ASD is sparse and yields mixed results, with some reports of preserved self-insight (76). By examining the links between subjective complaints and objectively measured impairments in the different components of social cognition, we wished to evaluate the self-consciousness of ASD adults with respect to their deficits in social functioning.

## Materials and Methods

The study was carried out in accordance with the Declaration of Helsinki and approved by the local Ethics Committee (CPP Lyon-Sud Est IV, no. 15/041; ANSM, no. 2015-A00580-49). Written informed consent to take part in the study was obtained from all participants. The control subjects were paid 30 euros for their participation.

### Participants

All participants were enrolled in a multisite study assessing social cognition in adults with autism and schizophrenia with the “ClaCoS” Battery. This study was conducted in three sites in France: Child Psychiatry Department specialized in autism, University Hospital of Tours, in Tours; Hospital Le Vinatier in Lyon and Groupe Hospitalier Universitaire Paris, Psychiatry and Neuroscience in Paris.

Participants with ASD without intellectual disability (*n* = 45), aged from 18 to 48 years were locally recruited and tested in Tours (*n* = 20), and Paris (*n* = 25). They were diagnosed by expert clinicians according to DSM-5 criteria (1) and using the Autism Diagnostic Observation Schedule, Second Edition [ADOS-2; (77)] and/or the Autism Diagnostic Interview-Revised [ADI-R; (78)]. All but three participants with ASD were screened with the ADI interview. The ADOS-2 was administered to twenty-two participants, including the three participants for which the ADI scores could not be obtained. ASD participants were either not under medication or on a stable medication regimen for a minimum of 1 month.

Typical developing adults (*n* = 45), aged from 18 to 50 years were recruited from the local community in Tours (*n* = 8), Paris (*n* = 5) and Lyon (*n* = 32). They were screened for the absence of any neuropsychiatric disorder using the MINI [International Neuropsychiatric Interview (79)].

Exclusion criteria for both groups included: [1] the presence or history of neurological disorders affecting brain function, [2] the presence of severe visual or hearing impairments interfering with assessment, [3] the absence of French language proficiency or important reading difficulties and [4] an abuse of substance in the past month (tobacco excluded).

The control participants were selected from a larger dataset (*n* = 200) to match the ASD population in terms of gender, age and education. The demographic and clinical characteristics of both groups are shown in Table 1. Verbal and Performance Intelligence quotients (IQ) were assessed in the ASD group by the Wechsler Adult Intelligence Scale-Fourth Edition [WAIS-IV; (80)] (Table 1).

**Table 1:** Participants characteristics.

	**Patients with ASD**		**Controls (***N*** = 45)**	***p***
	***N***	**Mean (SD)**	**Mean (SD)**	
Age (years)	45	27.7 (7.9)	27.6 (7.8)	0.968
Gender (F:M)	45	10:35	9:36	0.796
Education (years)	45	13.0 (2.4)	13.3 (1.7)	0.693
ADOS-2 (social interactions +communication)	22	12.6 (4.6)	–	NA
ADI (social interactions)	42	18.8 (7.9)	–	NA
ADI (verbal communication)	42	13.1 (5.3)	–	NA
Verbal IQ	45	117 (18)	–	NA
Performance IQ	45	103 (18)	–	NA
**Empathy quotient**	40	24.7 (10.5)	39.1 (10.4)	<0.001
**Rey tangled lines test**				
Time (ms)	44	9.8 (3.9)	7.9 (2.5)	0.004
Number of errors	44	1.0 (1.8)	0.6 (1.1)	0.691
**Cancellation task**				
Time (ms)	44	101.9 (47.2)	78.5 (24.8)	0.005
Number of errors	44	0.5 (0.9)	0.2 (0.5)	0.224

*Non-parametric Mann-Whitney or χ^2^ tests were used to test group differences*.

### Social Cognitive Measures

**Figure 1:**
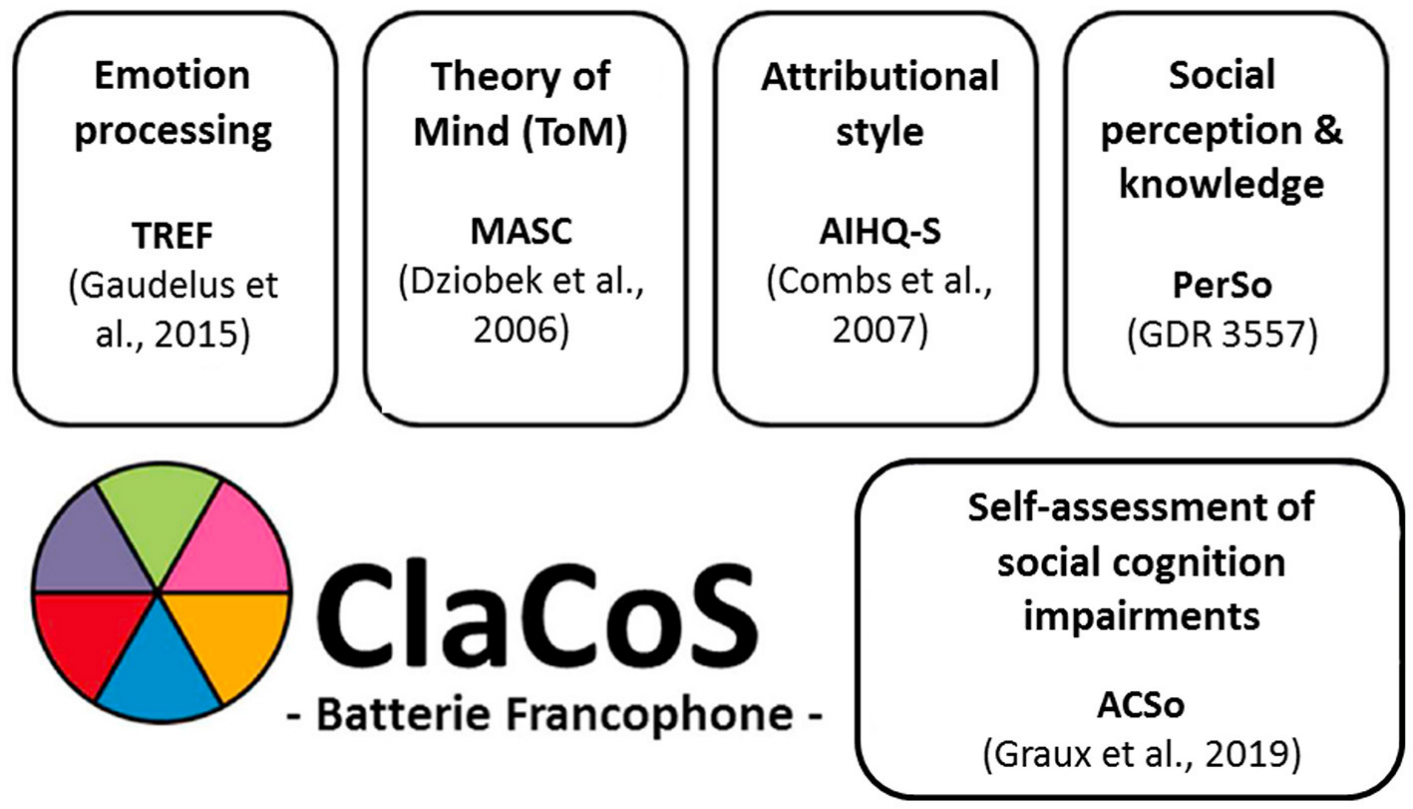
Tests constituting the “ClaCoS” battery.

All participants were tested in a silent room by an experienced neuropsychologist and placed, for computerized tests, at 23 inches from a 15-inch computer screen. They underwent a full assessment with the “ClaCoS” battery, developed by the multicentric Research group in psychiatry GDR3557 [for a more detailed presentation of each test, see (81)]. This new battery for social cognition evaluation included the following tests (Figure 1):

#### Self-Assessment of Social Cognition Impairments (ACSo)

The ACSo (82) is a self-administered questionnaire allowing to assess the subjective complaints of patients suffering from difficulties in the field of social cognition. Social cognition complaints are explored considering 4 dimensions: emotional perception, social perception and knowledge, theory of mind and attributional style. This allows the computation of 5 scores: a total score and 4 sub-scores corresponding to each domain.

#### Emotion Processing—Facial Emotion Recognition Test (TREF)

The TREF (83) assesses the ability to recognize five out of the six basic and universal emotions (happiness, anger, sadness, fear, and disgust). In addition, faces expressing contempt are presented, instead of surprise, as this more complex social emotion is of interest when considering psychiatric populations. Each emotion is presented with nine levels of intensity from 20 to 100%. The participants were asked to name the emotion expressed from the photos, using a forced choice among the six possible responses. This assessment provides an overall percentage of correct emotion recognition (global score) and for each emotion (score per emotion). Moreover, the level of intensity (global and per emotion) necessary for recognizing emotions with certainty was computed (recognition threshold). In this study, we specifically analyzed the global and per emotion percentage of correct recognition and recognition thresholds.

#### Social Perception and Knowledge—PerSo (GDR 3557)

The PerSo measures the perception of social situations, using pictures taken from the material “ColorCards—Social behavior.” Participants completed 3 successive tasks. First, they were asked to list all the elements perceived in the picture, providing a global “fluency score.” Then, they were instructed to explain the social situation freely, leading to a “non-indexed interpretation score.” Indexed questions were then proposed if some of the expected elements were missing (main character; location; interactions), resulting in an “indexed interpretation score.” A “total interpretation score” was then computed by adding the “non-indexed” and “indexed” interpretation scores. Finally, the participants were asked to extract a social rule that could be related to the card, producing a “social knowledge score.”

#### Theory of Mind (ToM)—Mental States Attribution—Movie for the Assessment of Social Cognition (MASC)

The MASC test [(55); French Translation (58)] is a video-based task measuring ToM abilities. It is a 15-min movie featuring four people meeting on a Saturday evening. The movie is regularly interrupted by a screen displaying a question referring to the actors' mental states (emotions or feelings, thoughts and intentions). Four scores are rated: a total score (correct “ToM” responses) and 3 scores according to error types: a “less ToM” score (“undermentalization” answers), a “no ToM” score (literal answers, with no mentalization), and finally an “excessive ToM” score (over interpretative response).

#### Attributional Style—Ambiguous Intentions and Hostility Questionnaire-Short Version (AIHQ-S)

AIHQ-S measures attributional biases from ambiguous social situations [AIHQ-S, Adapted From Combs et al. (63); French Version by Angelard et al. in Preparation]. The participants were instructed to read each situation and to picture themselves in these situations. They were then asked to answer several questions to measure three scores: [1] a hostility bias (HB), reflecting how hostile they rated the other's actions toward themselves; [2] an attribution of responsibility score which is the average of three ratings from Likert scales: an intentionality score—IS (whether the person acted on purpose); an anger score—AS (how angry it would make them feel) and a blame score—BS (how much they would blame the other person); [3] an aggression bias (AB) corresponding to the level of aggression in their response.

In addition, empathy was assessed using the Empathy Quotient questionnaire [EQ, (84)]. The Rey Tangled Lines Test (85) and a cancellation task (86) were also included as control measures of visual discrimination and visuo-spatial attention abilities. Due to technical reasons, data from the EQ was not recorded for five ASD participants and from both the Rey tangled lines test and the cancellation task for one ASD participant. All control participants completed these three additional tasks.

### Statistical Analysis

Statistical analyses were performed using STATISTICA v13.3 software (TIBICO^®^). For a better uniformity across the different analyses, non-parametric statistics were chosen, as there was a violation of the assumption of homogeneity of variance on some measures, according to the Levene's test. The Mann-Whitney non parametric *U* test was used for group comparisons. ANCOVAs were performed in order to control the effect of potential confounding variables. Relationships between subjective complaints and the neuropsychological measures were assessed with Spearman correlation analyses, considering the entire sample (ASD + controls). These were performed between each of the sub scores of the ACSo and the score obtained on the neuropsychological assessment of the corresponding dimension of social cognition (emotional perception, social perception and knowledge, theory of mind or attributional style), only when significant group comparisons were observed. Bonferonni correction for multiple analysis was applied.

## Results

### Demographic and Clinical Characteristics

The ASD and control groups did not differ in terms of age, gender or education level (Table 1). As expected, ASD individuals showed lower empathy scores compared to matched controls. They were also slower on both visuo-spatial and attentional tests, while their accuracy was similar to the control group.

### Comparative Results of Social Cognition Assessments in Adults With ASD and Controls

#### Self-Assessment of Social Cognition Impairments (ACSo)

The total score as well as all four sub-scores (emotional perception, social perception and knowledge, theory of mind and attributional style) were higher in adults with ASD compared to controls (all *p* < 0.001; Table 2).

**Table 2:** Scores from the “ClaCoS” battery in adults with ASD and controls.

	**ASD (** ***N*** **= 45)** **Mean (SD)**	**Controls (** ***N*** **= 45)** **Mean (SD)**	***U***	***p***	**Adjusted ***p*****
**Self-assessment of social cognition impairments (ACSo)**					
Total score	24.24 (8.0)	9.6 (5.1)	158.5	<0.001	<0.001
Emotional perception	3.5 (1.7)	1.3 (1.2)	293.0	<0.001	<0.001
Social perception and knowledge	6.4 (2.7)	2.5 (1.9)	262.0	<0.001	<0.001
Theory of mind	6.8 (2.4)	2.8 (1.7)	187.5	<0.001	<0.001
Attributional style	4.9 (2.8)	1.6 (1.4)	295.5	<0.001	<0.001
**TREF-Facial emotion recognition**					
% Of correct recognition	65.7 (10.2)	70.6 (6.4)	699.5	0.011	0.077
Happiness	87.2 (11.5)	91.1 (8.7)	925.0	0.468	3.276
Anger	61.1 (27.4)	68.1 (18.9)	909.5	0.406	2.842
Sadness	69.1 (19.6)	72.1 (19.2)	969.0	0.727	5.089
Fear	78.7 (15.7)	83.7 (13.5)	888.5	0.311	2.177
Disgust	57.7 (14.9)	62.2 (12.2)	933.5	0.522	3.654
Contempt	40.3 (21.9)	46.4 (17.9)	788.5	0.070	0.490
Recognition threshold	53.3 (9.9)	48.3 (6.6)	662.0	0.005	0.035
Happiness	32.3 (11.6)	29.4 (10.0)	886.5	0.291	2.037
Anger	58.6 (25.0)	50.2 (17.9)	800.5	0.086	0.602
Sadness	53.0 (18.9)	48.8 (16.8)	878.5	0.278	1.946
Fear	43.1 (16.0)	36.9 (14.6)	764.0	0.045	0.315
Disgust	58.1 (12.8)	55.0 (11.5)	858.5	0.206	1.442
Contempt	72.4 (17.8)	69.8 (13.6)	888.0	0.313	2.191
**PerSo-Social perception and knowledge**					
Fluency score	80.4 (36.9)	106.0 (31.7)	595.0	<0.001	0.004
Interpretation (total score)	19.2 (3.2)	20.9 (2.5)	703.0	0.012	0.060
Non-indexed interpretation	9.0 (2.0)	9.9 (1.7)	774.0	0.052	0.260
Indexed interpretation	10.2 (1.4)	11.0 (1.1)	660.5	0.003	0.015
Social knowledge score	4.8 (2.0)	5.6 (1.8)	770.5	0.048	0.240
**MASC-theory of mind**					
Total score	26.3 (5.4)	31.8 (3.6)	507.5	<0.001	<0.001
**Error types**					
Excessive ToM	7.2 (2.8)	5.1 (2.5)	688.0	0.008	0.032
Less ToM	7.7 (3.4)	6.1 (3.4)	808.5	0.098	0.392
No ToM	3.7 (2.1)	2.0 (1.7)	683.0	0.007	0.028
**AIHQ-S-Attributional style**					
Hostility bias	1.9 (0.7)	1.7 (0.6)	864.5	0.230	1.382
Attribution of responsibility score	2.8 (0.7)	2.4 (0.6)	694.0	0.010	0.062
Intentionality score-IS	3.1 (1.0)	2.6 (0.7)	659.5	0.004	0.026
Anger score- AS	2.3 (0.8)	2.1 (0.6)	811.5	0.104	0.626
Blame score- BS	2.8 (0.9)	2.5 (0.7)	771.0	0.051	0.307
Agression bias-AB	1.4 (0.4)	1.6 (0.4)	658.5	0.004	0.023

*Results of Mann-Whitney non parametric tests used for groups comparisons (statistical values U, p and adjusted p-values after Bonferonni correction for multiple analysis). Significant effects are highlighted in gray*.

#### Facial Emotion Recognition (TREF)

Adults with ASD required a higher threshold in order to correctly recognize emotional facial expressions from photographs (adjusted *p* = 0.035; Table 2). Moreover, a smaller recognition accuracy was observed with a trend in the ASD group compared to the controls (adjusted *p* = 0.077). This was found on the overall scores of correct recognition and recognition threshold but not when considering each emotion separately.

#### Social Perception and Knowledge (PerSo)

Adults with ASD were less efficient than control participants on the assessments of both the fluency and the interpretation of the social situation (Table 2). They listed fewer visual details from the perceptual scenes (fluency score; adjusted *p* = 0.004). Furthermore, the interpretation score was significantly lower in the ASD group compared to the controls when considering the indexed interpretation (adjusted *p* = 0.015) and with a trend for the total interpretation (adjusted *p* = 0.060).

#### Theory of Mind (MASC)

The total score (correct “ToM” answers) was higher in controls than in adults with ASD (adjusted *p* < 0.001; Table 2). More precisely, adults with ASD produced significantly more “no ToM” (adjusted *p* = 0.028) and “excessive ToM” (adjusted *p* = 0.032) answers compared to controls. No difference was found between adults with ASD and controls for the “less ToM” answer.

#### Attributional Style (AIHQ-S)

No difference was found between adults with ASD and controls for the hostility bias (Table 2). A significantly higher intentionality score (adjusted *p* = 0.026) was observed for adults with ASD compared to the controls, resulting in a trend for a greater attribution of responsibility score in the ASD group (adjusted *p* = 0.062). ASD and controls did not differ on the anger nor on the blame score. Moreover, the ASD participants showed a lower aggression bias than the controls (adjusted *p* = 0.023).

All differences previously reported between the ASD and controls remained significant after controlling for visuospatial discrimination and attentional abilities (all F > 6.20; *p* < 0.05).

### Relationship Between Subjective Complaints and Social Cognition Assessments

**Figure 2:**
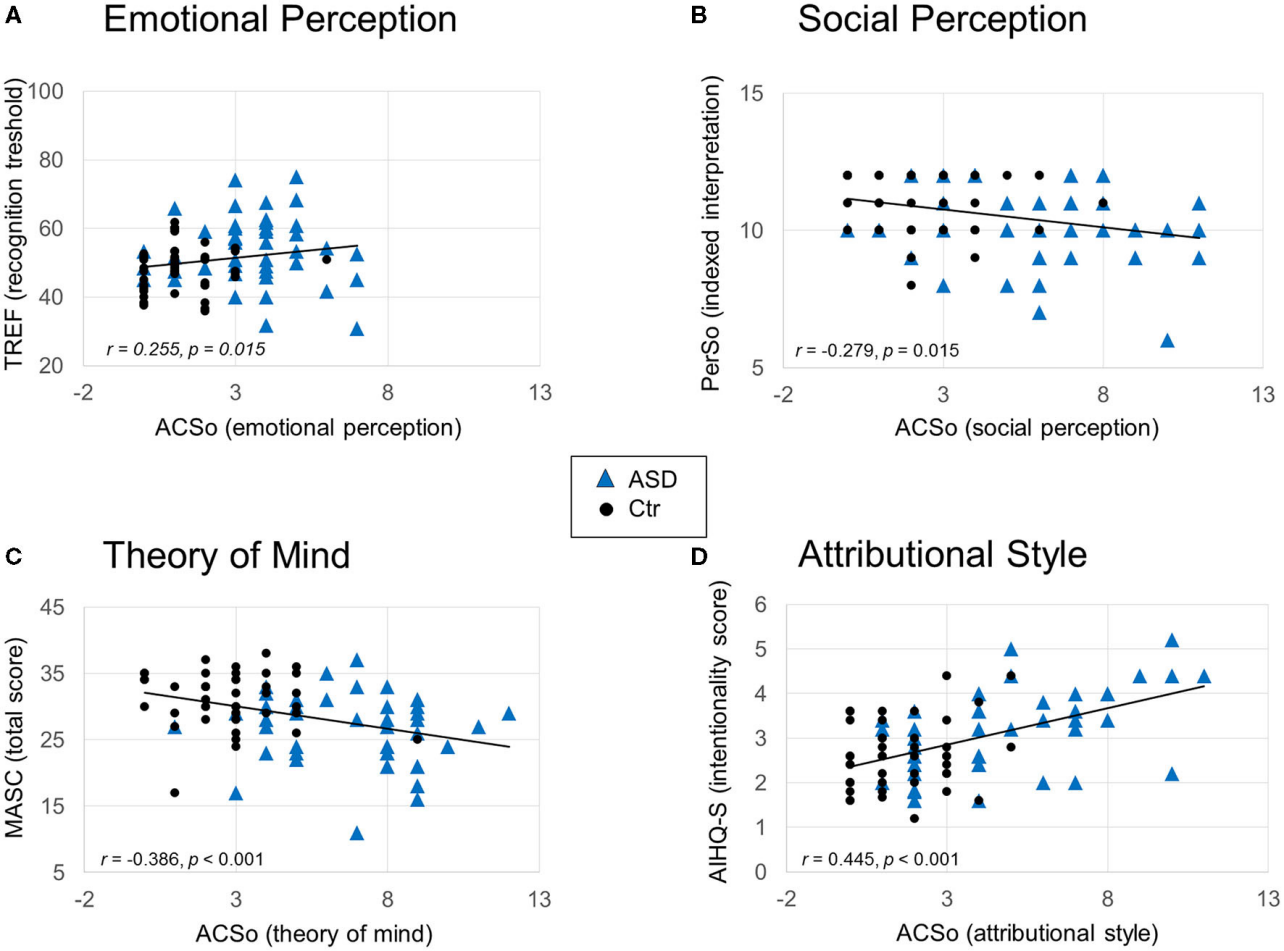
Main correlations between subjective complaints and social cognition assessments for each of the four domains. Results of Spearman correlations are reported (statistical values r and adjusted p after Bonferonni correction for multiple analysis). **(A)** Emotional perception complaint plotted against the recognition threshold of the TREF. **(B)** Social perception and knowledge complaint plotted against the indexed interpretation score of the PerSo. **(C)** Theory of mind complaint plotted against the total score of the Masc. **(D)** Attributional style complaint plotted against the intentionality score of the AIHQ-S. The solid lines represent the linear regressions.

Correlations were performed between the scores of each dimension of the ACSo and the results on the test designed to assess social cognitive impairments on the corresponding dimension of social cognition. This allowed us to examine the relationship between the participant's subjective complaints in specific domains of social cognition and the actual impairments measured through the neuropsychological assessment (Figure 2).

#### Facial Emotion Recognition (Relationship Between ACSo- Emotional Perception and TREF)

Subjective complaint concerning emotional perception (ACSo) was positively correlated with the emotion recognition threshold (TREF threshold; *r* = 0.255, *p* = 0.015). Thus, greater complaints concerning emotional perception were associated with overall higher thresholds needed in order to correctly recognize emotional facial expressions.

#### Social Perception and Knowledge (Relationship Between ACSo- Social Perception & PerSo)

Subjective complaint concerning social perception and knowledge (ACSo) was negatively correlated with the interpretation score (PerSo indexed interpretation score: *r* = −0.279, *p* = 0.008, adjusted *p* = 0.015). Subjective complaint concerning social perception and knowledge (ACSo) was not correlated with the fluency score (PerSo fluency, *r* = −0.115, *p* = 0.280).

#### Theory of Mind (Relationship Between ACSo- Theory of Mind and MASC)

Subjective complaint concerning theory of mind (ACSo) was negatively correlated with the total score (MASC; *r* = −0.386, *p* < 0.001, adjusted p < 0.001). Moreover, it was positively correlated with the “no ToM”(*r* = 0.253, *p* = 0.016, adjusted p = 0.048) and with a trend to “excessive ToM” (*r* = 0.243, *p* = 0.021, adjusted *p* = 0.063) answers. The larger the complaint, the smaller the total score and the greater the “no ToM” and “excessive ToM” answers.

#### Attributional Style (Relationship Between ACSo- Attributional Style and AIHQ-S)

Subjective complaint concerning attributional style (ACSo) was positively correlated with the intentionality score (*r* = 0.445, *p* < 0.001, adjusted *p* < 0.001). Thus, larger complaints were associated with a higher attribution of the other's responsibility. No significant correlation was observed between subjective complaint concerning attributional style and the aggression bias (*r* = −0.061, *p* = 0.568).

## Discussion

The main goal of our study was to evaluate the relevance of the “ClaCoS” battery for the assessment of social cognition impairments in adults with ASD without intellectual disability. We further examined the links between subjective complaints and objectively measured impairments on the different components of social cognition. Overall, adults with ASD reported greater subjective complaints than controls in each of the four areas explored by the ACSo. They also showed deficits on all of the neuropsychological tests from the “ClaCoS” battery, which explored the same four main components of social cognition: emotional perception, social perception and knowledge, theory of mind and attributional style (70, 81). Moreover, each of the four sub-scores of the ACSo were correlated with the performance on the corresponding social cognitive assessment.

### Differences Between ASD and Controls on Objective Evaluations

Adults with ASD were impaired in all four domains of social cognition assessed by the “ClaCoS” battery. Deficits in emotional perception and theory of mind are classically reported in ASD, while social perception and attributional style are less frequently explored. In particular, deficits in emotional perception and theory of mind constitute core features of ASD and are crucial clinical signs examined in the process of diagnosing ASD (1). However, classical social cognition tests often fail to show such deficits in adults with ASD without intellectual disability, due to a lack of sensitivity in this specific population [(7, 35, 51, 52); for a meta-analysis, see (15)]. Our results thus confirm that more challenging and ecological tests presenting either subtle expressions (i.e., the TREF) or ecologically valid and dynamic stimuli (i.e., the MASC) are particularly interesting tools in order to assess emotional perception and theory of mind impairments, respectively, in ASD adults without intellectual disability.

Using the TREF, in the ASD group compared to the controls, we observed a higher global recognition threshold and a trend for a lesser overall percentage of correct emotion recognition. Thus, the recognition threshold seems to be a more relevant measure in order to uncover impairments on emotional recognition in this population. Our result is consistent with an extensive amount of researches showing impairments on emotional processing in ASD [(9); for a meta-analysis, see (15)]. Moreover, this was observed as a global effect and was not related to a specific emotion. Taken together our results support the hypothesis of a broad impairment of emotion recognition in individuals with ASD [for a review, see (14)].

Adults with ASD showed lower scores at the PerSo compared to controls, suggesting impaired social perception. More precisely, they reported fewer visual elements from the social scenes (fluency score) and were less accurate in their interpretation of the depicted social situations. The difference between groups on the number of visual elements reported does not seem to be exclusively linked to slower overall visuospatial or attentional abilities in ASD. Indeed, it remained significant when controlling for such effect of visual attention. It could rather reflect slower overall cognitive processing or more specifically slower verbal elaboration required in this task. It could also reflect a well-described lack of generation of new ideas (87–89). Concerning the interpretation scores, the indexed interpretation was significantly different between the two groups and this difference was observed with a trend for the total interpretation. This suggests that adults with ASD respond similarly as the typical developing controls when asked to freely describe the depicted social situation from pictures. However, they were less facilitated by the indexed question compared to controls, in line with a recent meta-analysis (40). Altogether, our study shows difficulties in the ASD group, in the identification of the main contextual visual elements which allow to understand social situations (i.e., location; main characters and interactions between these characters as well as emotions expressed). Although the term “social perception” is not common in the field of ASD research, eye-tracking studies reveal impaired attention toward social compared to non-social visual stimuli [(38, 39); for meta-analyses, see (40, 41)]. The lack of visual processing, and binding of the salient social and non-social contextual elements are likely to contribute to difficulties encountered by ASD individuals in understanding and thus adjusting to social situations. Nevertheless, the ASD individuals were able to identify social rules which suggests an absence of a general deficit in social knowledge *per se*. Indeed, ASD individuals may learn social rules, although in a somewhat rigid manner. However, they often encounter greater difficulties to conveniently apply these rules in everyday life (90). Hence, the PerSo constitutes an interesting tool to assess social perception and knowledge in ASD. To our knowledge, the PerSo is the only existing standardized tool that examines this component of social cognition, at least in French language.

As expected, we observed an impaired theory of mind (ToM), in line with previous studies showing that the MASC test is sensitive to reveal subtle ToM alterations in adults with ASD, without intellectual disability (55–60). Interestingly, our study shows two main types of errors in the ASD group compared to controls: greater “Excessive ToM” and “No ToM.” Previous studies showed that ASD individuals made these types of errors more frequently than controls (“Excessive ToM,” “Less-ToM,” and “No ToM”) (56–58). Moreover, these studies reported inconsistent findings, with either prominent over-mentalizing (excess) or under-mentalizing (less) errors, while concrete cognition (No) was systematically less frequent, in accordance with our study. The absence of difference between groups on the “Less ToM” errors in the present study and more generally the discrepancy across studies may depend upon sociodemographic specificities (e.g., education level, age, gender…). Our study supports the hypothesis of two different types of ToM impairments in ASD, with either an overmentalizing or an undermentalizing tendency (58). The concept of hyper- and hypo-mentalization has been introduced in schizophrenia research (91), some authors suggesting the existence of a continuum from hypo- to hyper- ToM, across different psychiatric illnesses (92). Further studies including patients with other psychiatric disorders such as schizophrenia are needed in order to test this hypothesis. This approach fits with a transnosographic view of common symptomatic profiles between patients with ASD and schizophrenia.

To date, there are very few studies focusing on attribution biases in ASD. This component of social cognition is rarely explored in clinical practice. The studies mainly focused on causal attribution (internal vs. external) and showed no differences on attributional style between ASD adolescents and adults compared to neurotypical individuals (47–49, 75). However, the present study specifically considers hostility and aggression biases as well as the different sub-dimensions leading to attribution of responsibility. Our results revealed higher attribution of responsibility (specific to the intentionality of other's actions) and lower aggression bias in the ASD group compared to controls, while no difference was observed on the hostility bias. Thus, adults with ASD without intellectual disability tend to consider other's actions as being more surely intentional in ambiguous social situations but mostly do not show paranoid ideas. They don't perceive other's actions in these ambiguous situations as particularly hostile toward themselves, do not experience more anger and do not blame others more than the controls do. They further tend to respond in a passive way, as illustrated by the lower aggression bias compared to controls. These results are consistent with the clinical observations of rigid thought and tendency to systemize in ASD individuals, which may explain the higher attribution of intentionality score (93). The AIHQ-S thus seems an interesting tool to help clinicians to separate ASD from other psychiatric disorders, particularly schizophrenia. Indeed, these two conditions can be difficult to distinguish in adult patients, with a risk of misdiagnosis (94). Paranoid ideations in ASD and in schizophrenia are a consequence of different mechanisms. Persecutory delusions are commonly seen in people with schizophrenia. However, paranoïd ideations observed in adults with ASD are generally linked to the negative social experiences (e.g., being teased, bullied, or rejected), or to the specific cognitive style of this population (rigid thought, focusing on details, alteration in ToM or in social perception) (47, 48). This can lead to interpretation biases, generally associated with behavioral reactions of avoidance and social isolation rather than with overtly aggressive behaviors (75). The “ClaCoS” should however be used with caution for purposes of differential diagnosis, in light of the frequent comorbid psychiatric conditions associated with ASD [e.g., ADHD, bipolar disorder, anxiety disorder…; for a review, see (95)]. Future studies including data of other population of patients are required in this perspective.

### Links Between Subjective and Objective Evaluations

An interesting feature of the “ClaCoS” is the assessment of patients' subjective complaints in daily life in the field of social cognition. To our knowledge, the ACSo is the first transnosographic scale examining this question. Our results suggest that adults with ASD without intellectual disability are able to express complaints from a self-report questionnaire. This short scale seems to constitute an interesting media which may help ASD adults to express the challenges they encounter in social situations, especially for those having difficulties in verbal expression or initiation. Interestingly, there was an association between subjective complaints and objective measures obtained from the neuropsychological assessment in all four domains of social cognition. These results tend to support the relevance of each neuropsychological test of the “ClaCoS” battery to assess the specific domain of social cognition which impacts the participant's everyday social functioning. They further suggest that ASD adults are aware of their social difficulties in different areas, in line with their good insight and metacognitive abilities (76, 96). In the validation study of the ACSo, Graux et al. (82) did not observe any correlation between objective and subjective assessments of social cognition components (emotional perception and theory of mind), suggesting an altered “social cognitive insight” in adults with psychiatric disorders. However, in a transnosographic perspective, statistical analyses were performed in patients with different diagnoses, including a majority of participants with schizophrenia and a limited number of participants with ASD.

### Control Analyses and Limitations

It seems unlikely that our effects were mediated by socio-demographic factors, as the groups were matched according to age, gender, and educational level. Moreover, the ASD group had good intellectual and very efficient language abilities, as revealed by their IQ measures, suggesting that the deficits observed in the evaluation of social cognition are unlikely to be linked to poorer intellectual or verbal abilities. Note however that we could not fully control the impact of verbal and performance IQ, as these were not recorded in control participants. Although the ASD participants were slower on both visuo-spatial and attentional tests, they correctly processed the visual information. They were sufficiently engaged in the task but needed more processing time, consistently with previous observations (97, 98). Furthermore, all group differences remained significant when controlling for visual attention. Thus, the lower performances on social cognition tests in the ASD group cannot be exclusively explained by slower visuo-spatial attention and exploration abilities.

Our results should nevertheless be interpreted with several limitations. Although our population was larger than most studies considering the assessment of social cognition in ASD, it remains of a moderate sample size. Future studies including larger groups are needed in order to replicate these findings. In particular, the correlations reported here were of medium effect size (Cohen criterion) and thus have to be taken with caution. Moreover, the ASD participants had average or over-average Intellectual Quotients. Their verbal IQ was in average higher than the performance IQ. Future studies are required to evaluate whether these results can be generalized to ASD individuals with lower verbal or general intellectual abilities. More generally, the links between neurocognitive and social cognitive performance remains to be explored. The addition of other validated tools for social cognition evaluation could also be interesting as external validation of the tests constituting the “ClaCoS” battery. Furthermore, the use of clinical tools allowing the assessment of hyper- and hypo-mentalization in ASD could allow the identification of different clinical profiles, in line with the transnosographic view of psychiatric illnesses. Further studies including larger sample size and different clinical profiles are needed in order to confirm and extend the present results.

## Conclusion

The “ClaCoS” is a functional evaluation battery of the four main dimensions of social cognition which may be altered in different psychiatric conditions, consistent with a transnosographic perspective. To our knowledge, “ClaCoS” is the only existing social cognitive battery including a subjective evaluation of the individual's impairment, as well as an assessment of attributional style. As a whole, the current study suggests that performance on the “ClaCoS” battery accurately reflects everyday life difficulties of adults with ASD. It seems to be a well-suited tool to help uncover alterations in specific domains of social cognition in this population. This allows the selection of the most appropriate therapeutic program according to each patient's functional profile: from the most basic perceptual processes to higher level metacognition abilities. Our study shows that ASD adults without intellectual disability have a good self-awareness of their impairments in different domains of social cognition which can lead to the specific challenges they encounter with social functioning in everyday life situation. Self-assessment helps to involve and motivate patients to participate in cognitive remediation therapy (99) and the effectiveness of these therapies relies in part on the patients' awareness of their disorders. Thus, our results are a good prognostic indicator for the engagement of ASD individuals in cognitive remediation programs. The “ClaCoS” battery further provides interesting new elements which may be contributive in a diagnostic procedure, alongside the classical tools and clinical evaluations, in a dimensional and lifelong evolving perspective of ASD.

## Data Availability Statement

The raw data supporting the conclusions of this article will be made available by the authors, without undue reservation.

## Ethics Statement

The studies involving human participants were reviewed and approved by CPP Lyon Sud Est IV, No. 15/041; ANSM, No. 2015-A00580-49. The patients/participants provided their written informed consent to participate in this study.

## Author Contributions

SM-K, AT, LB, CD-A, EP, ZP, IC-M, BG, IA, JG, and EH-D made substantial contributions to conception and design. SM-K, AT, LB, CD-A, EP, ZP, IA, JG, and EH-D made substantial contributions to acquisition of data. SM-K, AT, SR, JG, and EH-D made substantial contributions to analysis and interpretation of data. SM-K, AT, LB, CD-A, EP, ZP, SR, IA, JG, EH-D, IC-M, BG, M-OK, NF, and FB-B participated in drafting the article. All authors contributed to, have approved the final manuscript and agree to be accountable for the content of the work.

## Conflict of Interest

The authors declare that the research was conducted in the absence of any commercial or financial relationships that could be construed as a potential conflict of interest.

## Publisher's Note

All claims expressed in this article are solely those of the authors and do not necessarily represent those of their affiliated organizations, or those of the publisher, the editors and the reviewers. Any product that may be evaluated in this article, or claim that may be made by its manufacturer, is not guaranteed or endorsed by the publisher.
